# Comparison of Long-Term Effect of Dual-Chamber Pacing and Alcohol Septal Ablation in Patients with Hypertrophic Obstructive Cardiomyopathy

**DOI:** 10.1155/2013/629650

**Published:** 2013-11-11

**Authors:** Jan Krejci, Pavel Gregor, David Zemanek, Klaudia Vyskocilova, Karol Curila, Radka Stepanova, Miroslav Novak, Ladislav Groch, Josef Veselka

**Affiliations:** ^1^Department of Cardiovascular Diseases, St. Anne's University Hospital—International Clinical Research Center and Masaryk University, Pekařská 53, 656 91 Brno, Czech Republic; ^2^Cardiocenter, 3rd Department of Internal Medicine—Cardiology, University Hospital Kralovske Vinohrady and 3rd Medical School of Charles University, Šrobárova 1150/50, 100 34 Prague, Czech Republic; ^3^Department of Cardiology, 2nd Medical School of Charles University, University Hospital Motol, V Úvalu 84, 150 06 Prague, Czech Republic; ^4^International Clinical Research Center, St. Anne's University Hospital, Pekařská 53, 656 91 Brno, Czech Republic

## Abstract

*Introduction*. Nonpharmacological treatment of patients with hypertrophic obstructive cardiomyopathy (HOCM) comprises surgical myectomy (SME), alcohol septal ablation (ASA), and dual-chamber (DDD) pacing. The aim of the study was to compare the long-term effect of DDD pacing and ASA in symptomatic HOCM patients. *Patients and Methods*. We evaluated retrospective data from three cardiocenters; there were 24 patients treated with DDD pacing included and 52 treated with ASA followed for 101 ± 49 and 87 ± 23 months, respectively. *Results*. In the group treated with DDD pacing, the left ventricle outflow tract gradient (LVOTG) decreased from 82 ± 44 mmHg to 21 ± 21 mmHg, and NYHA class improved from 2.7 ± 0.5 to 2.1 ± 0.6 (both *P* < 0.001). In the ASA-treated group, a decline in LVOTG from 73 ± 38 mmHg to 24 ± 26 mmHg and reduction in NYHA class from 2.8 ± 0.5 to 1.7 ± 0.8 were observed (both *P* < 0.001). The LVOTG change was similar in both groups (*P* = 0.264), and symptoms were more affected by ASA (*P* = 0.001). *Conclusion*. ASA and DDD pacing were similarly effective in reducing LVOTG. The symptoms improvement was more expressed in patients treated with ASA.

## 1. Introduction

Hypertrophic cardiomyopathy (HCM) is the most frequent cardiomyopathy with the prevalence of 0.2% [1]. HCM is characterized by the presence of left ventricular wall hypertrophy, which does not have an alternative cause. It was first described more than half a century ago [2, 3]. Leaving aside HCM phenocopies, the inheritance of HCM is autosomal dominant, and the mutations affect primarily the genes for sarcomeric proteins [4]. Patients with HCM can be divided into three similarly sized groups according to the presence or absence of left ventricle outflow tract gradient (LVOTG)—patients with resting obstruction, patients with latent obstruction occurring after provocation, and patients without obstruction [5]. The presence of significant pressure gradient (above 30 mmHg) was identified as a predictor of adverse prognosis [6]. Obstruction is highly variable and is influenced by a number of physiological situations [7]. The presence of LVOTG was, in the “preechocardiographic” era, an essential feature of patients with HCM [8]. LVOTG therefore became also the first target of therapeutic efforts. The first method of treatment used in this area at the beginning of the 1960s was surgical myectomy (SME) [9, 10]. In the 1970s, the possibility of reducing LVOTG by dual-chamber (DDD) pacing with apical preexcitation was described [11]. This method enjoyed great expansion in the 1990s, when a number of papers [12–14] documenting significant decrease of LVOTG and concomitant improvement in the functional status of patients with hypertrophic obstructive cardiomyopathy (HOCM) after DDD pacing were published. Cooling of interest in this method was linked to studies from the late 1990s, which however evaluated only the short-term effect of pacing [15–17].

In 1995, another alternative to surgical treatment of obstructive HCM was introduced. The method was originally called nonsurgical septal reduction of myocardial hypertrophy, which currently is known as alcohol septal ablation (ASA) [18]. This approach became the most frequent non-pharmacological treatment of HOCM resistant to optimal pharmacological therapy. It is estimated that since 1995, more than 5000 of these procedures have been performed, which exceeds the number of SMEs performed for more than 45 years [19, 20].

While surgical treatment is still the method of choice in a number of centers mainly in the US [5], in most European countries, ASA clearly prevails. Pacing is currently used only rather rarely. In recent years, several studies showing a very good long-term effect of pacing have been published [21–24]. At the same time, the question arose as to whether the negative attitude to this treatment approach is justified and whether it should not be reevaluated [25].

The aim of our study was to compare the long-term effect of ASA and DDD pacing on the clinical status and echocardiographic parameters in patients with HOCM.

## 2. Materials and Methods

### 2.1. Patients

This retrospective nonrandomized study included symptomatic patients (NYHA class III-IV or exertional syncope) with significant resting LVOTG despite an established optimal pharmacological treatment. In the period before the introduction of ASA into the therapeutic armamantarium all these patients were treated by DDD pacing. After the introduction of ASA into the treatment of patients with HOCM this became the primary non-pharmacological therapeutic approach. The patients were divided into two groups; the first group included patients treated by DDD pacing and the second group patients treated by ASA. The DDD pacing group consisted of 24 patients, followed for 101 ± 49 months (median 110 months); NYHA class was 2.7 ± 0.5, and resting LVOTG was 82 ± 44 mmHg before pacemaker (PM) implantation. The ASA treated group consisted of 52 patients, monitored for 87 ± 23 months (median 80 months), NYHA 2.8 ± 0.5, and baseline LVOTG 73 ± 38 mmHg. The two groups did not differ significantly in any of the baseline parameters except for left ventricle ejection fraction (LVEF) and LV diastolic diameter (Dd) (see Table 1).

### 2.2. Protocol

Data from the registers of three individual institutions were collected. The data relating to ASA were entered prospectively, while the data on patients treated with DDD pacing were obtained mostly retrospectively (with the exception of 4 patients who did not undergo ASA due to inappropriate coronary morphology or who refused this approach and preferred DDD pacing). The study was approved by local ethics committees. The diagnosis of HCM was based on echocardiographic assessment according to recommendations in HCM Guidelines [5, 26]. A limit of the left ventricle (LV) myocardial wall hypertrophy required for classification as a diagnosis HCM was 15 mm. The HOCM patients with LVOTG higher than 30 mmHg at rest assessed by continuous wave Doppler echocardiography and symptoms of NYHA III and IV resistant to optimal pharmacologic therapy or patients with exertional syncope, high LVOTG, and lower NYHA classification were regarded as candidates for nonpharmacologic therapy. 

All patients treated by DDD pacing were included in the evaluation, if documentation was sufficient enough and required parameters were accessible. Only patients after ASA followed up for more than 5 years were included into the study in order to achieve a comparable length of follow-up in both groups. Thus, the patients with reintervention (re-ASA), the emergence of AVB requiring permanent pacing, death, and with followup of less than 5 years were excluded. Changes in functional status assessed by NYHA classification and evolution of some echocardiographic parameters (especially LVOTG) in each treatment group were evaluated, and these results of both groups were then compared.

PM implantation was performed in a standard way with the introduction of the ventricular electrode tip consistently in the apex of the right ventricle (RV). Atrioventricular (AV) delay was set under ECG control to ensure full capture stimulation without the presence of spontaneous or fused contractions. In most patients, AV intervals were optimized under echocardiographic control so that LVOTG was reduced, while the stroke volume was not significantly affected.

ASA was conducted in the usual manner by application of small amount of alcohol (1–4 mL) into one of the septal branches of the left anterior descending coronary artery. The appropriate septal branch was selected after application of the echo-contrast substance into septal branch, and exclusion of perfusion to other areas of LV or RV was always carefully monitored by transthoracic echocardiography. Temporary transvenous pacing was introduced in all patients undergoing ASA because of the possible emergence of complete AV block during procedure. Continuous simultaneous monitoring of the pressures in LV apex and LVOT was carried by two separate catheters.

### 2.3. Statistical Analysis

The parameters were analyzed descriptively, and for the presentation of data, mean, standard deviation and median with a 95% confidence interval were used. Because the assumption of normal distribution was violated for most parameters (Shapiro-Wilk's test), nonparametric analyses were performed. For comparison of parameters before and after treatment, the Wilcoxon signed-rank test was conducted. For the comparison of parameters between the groups with different treatment, the Mann-Whitney test was performed. Results with a *P* value <0.05 were considered statistically significant.

## 3. Results

**Figure 1:**
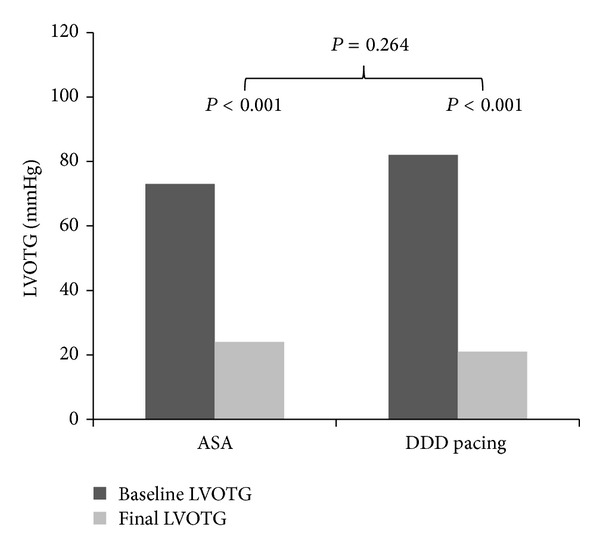
ASA—alcohol septal ablation, DDD pacing—dual chamber pacing, and LVOTG—left ventricle outflow tract gradient.

**Figure 2:**
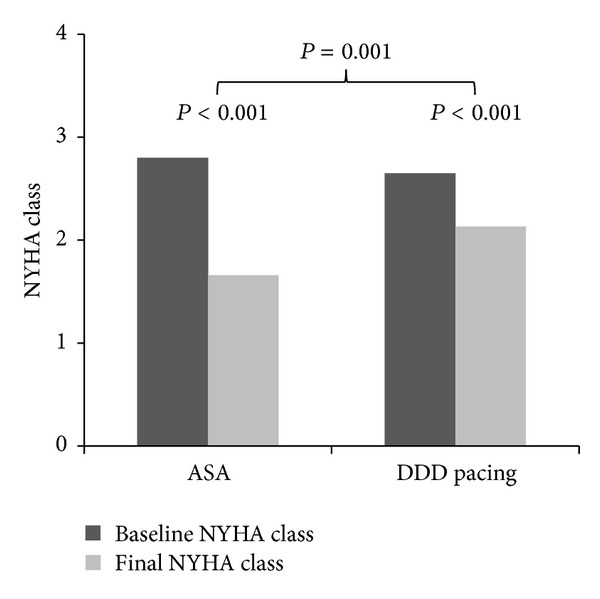
ASA—alcohol septal ablation and DDD pacing—dual chamber pacing.

In the group treated by DDD pacing, LVOTG decreased by 61 ± 48 mmHg (from 82 ± 44 to 21 ± 21 mmHg), NYHA class improved from 2.7 ± 0.5 to 2.1 ± 0.6 (both *P* < 0.001), and interventricular septum (IVS) thickness and LVEF were reduced (both *P* = 0.001). In the ASA group, LVOTG dropped by 49 ± 34 mmHg (from 73 ± 38 to 24 ± 26 mmHg) and NYHA from 2.8 ± 0.5 to 1.7 ± 0.8 (both *P* < 0.001). These results are illustrated in Figures 1 and 2. Basal septum thickness and left ventricle ejection fraction (LVEF) decreased (both *P* = 0.001) and LV Dd increased (*P* < 0.001) in the ASA treated group. When comparing the effect of treatment in both groups, no significant difference in the change of LVOTG and LVEF between ASA a DDD pacing group was observed. In the ASA group, more pronounced NYHA class improvement, reduction in the thickness of IVS (both *P* < 0.001), and LV Dd increase (*P* < 0.01) were observed (details in Table 1). No patients have undergone SME or cardiac transplantation, single-lead cardioverter/defibrillator (ICD) was implanted in 3 patients in the ASA group for primary prevention of sudden cardiac death. Pacemaker implantation was needed in one patient because of atrial fibrillation and bradycardia 7 years after ASA.

## 4. Discussion

In this retrospective study, we decided to analyze data from three cardiocenters which are engaged in two non-pharmacological treatment approaches for obstructive HCM-DDD pacing and ASA. 

There are only a few studies comparing pacing with other nonpharmacological treatments [27–30], so we decided to compare the effect of ASA and DDD pacing during long-term monitoring. The uniqueness of this work lies in the long and similar followup in both compared groups—more than 7 years (ASA group) and more than 8 years (DDD pacing group). Our results support the view that ASA leads to a sustained long-term decline of the LVOTG and to improvement of symptoms. They indicate that long-term DDD pacing with apical preexcitation results in a comparable reduction LVOTG as well, and also to improvement of the functional status which is, however, less pronounced than in the case of ASA.

Although SME is still considered to be the gold standard therapy, ASA is, especially in European countries, the most frequently performed intervention [19]. The method itself underwent development for nearly two decades; periprocedural contrast echocardiography is routinely used to select the appropriate septal branch and to avoid collateral myocardial perfusion. Also, smaller volume of alcohol is administered, leading to a lower incidence of AVB while maintaining hemodynamic and clinical effect [20, 31–34].

PM implantation is a routine procedure for several decades. The very important issue is the programming of the PM which requires careful and specific setting. In order to achieve the optimal effect, it is necessary to select and reevaluate the appropriate device setting, especially in patients with an unsatisfactory therapeutic response [22, 24]. 

The way the DDD pacing reduces LVOTG is not entirely clear. It can be caused by contribution of several mechanisms—inverse activation of IVS, IVS paradoxical movement, and some reported reduction of myocardial contractility induced by pacing as a very important aspect. All of this together leads to the enlargement the LV outflow tract, decrease of systolic anterior motion, and decline in LVOTG [14, 15, 24, 25, 35]. The acute pacing effect differs from the long-term; long-term stimulation induced dyssychrony leads to remodeling of the LV, which is responsible for the long-term decline of LVOTG enhanced with continued treatment. The time needed to induce the remodeling is probably longer than 12 months [23, 36]. This fact is the basic drawback of PIC [16] and M-PATHY [17] randomized trials and also of a study from the Mayo Clinic [15], whose design comprised 3-month alternative inactive (AAI 30/min) and active (DDD) pacing. 

Initial enthusiasm for pacing as a less invasive alternative to SME was followed by skepticism after publication of the results of these studies. All of these studies (PIC, M-PATHY, and Nishimura's study) had virtually identical crossover design, where insufficient length of active treatment could not lead to a fully expressed effect of pacing. The results of the largest of these studies, the PIC trial, have demonstrated a statistically significant decrease of LVOTG, as well as an improvement in the clinical status of patients on active treatment [16, 37]. Despite the fact that PM implantation certainly has a placebo effect, reduction of LVOTG was more expressed in the treated group (DDD pacing) than in patients with inactive pacemaker settings [38]. Furthermore, in another related study by Gadler, 10 patients already treated for 19 ± 4 months by DDD pacing were randomized to an inactive AAI pacing mode or to continue in active DDD stimulation. Due to the recurrence of symptoms and an increase in LVOTG, patients randomized to inactive treatment had to be reprogrammed early into the DDD paced group, with the rapid retreat of symptoms and decrease LVOTG [39]. The results of M-PATHY were not so favorable, but they also showed a significant LVOTG decrease [17]. Reflection of M-PATHY however has led to the fact that the pacing was almost abandoned, and the Guidelines published in 2011 attributed entirely marginal significance to this therapeutic option [5]. 

Clinical experience from long-term monitoring of patients treated with DDD pacing, as well as the shortcomings of the studies mentioned above, has led some authors to attempt to review the status of cardiac pacing in the treatment of obstructive HCM, especially with regard to its long-term effect, which has not been evaluated in these studies at all [21–24].

Already, studies by Jeanrenaud and Fananapazir from the 1990s showed that the effect of pacing on LVOTG increases with time [13, 36]. A more recent study by Megevand et al. documented a decline in LVOTG after pacing from the initial 82 ± 35 mmHg to 42 ± 33 mmHg after 4 months of treatment. The decline continued, so that after an average of 49 months of treatment, LVOTG was reduced to 32 ± 23 mmHg [21]. The next paper showing a steady decline in LVOTG after long-term pacing was published by Topilski et al. In addition to LVOTG decline from 91.8 ± 28.2 mmHg to 22.8 ± 28.1 mmHg, this study documented decrease in NYHA class from 3.1 ± 0.7 to 1.3 ± 0.4 after an average of 68 months of pacing [22]. A very important study in this area was the study by Galve et al, which monitored 50 patients for an average of 5 years and demonstrated progressive LVOTG decrease with time, from baseline 86 ± 29 mmHg LVOTG fell in the 3rd month to 55 ± 37 mmHg, which corresponds to the decrease achieved in the studies PIC and M-PATHY for the same treatment period. One year after the implantation, LVOTG further decreased to 41 ± 26 mmHg and at the final evaluation even to 28 ± 23 mmHg (*P* = 0.0001). Significant improvement in the NYHA classification and distance extension during 6-minute walk test was also documented [23]. An additional study published by Sandín et al. showed a significant LVOTG decrease after PM implantation. Also here, as in previous studies, more pronounced decrease of LVOTG at the final examination was found than at the first control carried out within 1 year after implantation [24].

Results of our study support the hypothesis of long-term progressive LVOTG decline after pacemaker treatment. The LVOTG decrease after ASA is certainly more rapid, but after several years, the effect of DDD pacing on LVOTG is similar to that of ASA. Functional improvement in our group of pacemaker treated patients was less pronounced than for ASA. The probable key point may be improvement of diastolic function. ASA results not only to the reduction of IVS thickness but also to decrease in LV mass, which may improve LV diastolic function [40]. This very likely contributes (in addition to the actual LVOTG decline) to the observed functional improvements in this treatment. Conversely, RV pacing worsens LV diastolic function, especially in the case of preexisting diastolic dysfunction which is a typical feature of HCM [41]. Moreover, apical RV pacing also negatively affects LV systolic function, as demonstrated by comparison with biventricular pacing for patients with normal LV systolic function indicated for bradycardia in the PACE study [42]. Other authors also describe an increased incidence of heart failure and LV ejection fraction decrease in RV pacing [43]. In our study as well, LVEF decline was more pronounced in patients treated with pacing than in patients treated with ASA, although the difference did not reach statistical significance. 

Bearing in mind a number of limitations of this study, our results suggest that both treatment modalities may be equally effective in reducing LVOTG. Symptoms improvement was also evident in both treatments but was statistically more significant in patients treated with ASA. Positive LV remodeling was observed only after ASA; also, reduction of IVS hypertrophy was more pronounced in patients treated with ASA. DDD pacing probably will not be first choice therapy, but although these assumptions need to be confirmed by further studies, our results suggest that DDD pacing could be considered as one of the treatment options in HOCM, especially in elderly patients. The task of the physician is to select the optimal method of treatment for each individual patient, taking into account actual heart morphology with the extent and localization of hypertrophy, coronary and valvular morphology, age and comorbidities, availability of procedures, the presence of conduction disturbance, and the risk of arrhythmia or sudden cardiac death requiring ICD implantation and, not least, to consider the patient's decision [25, 35]. 

### 4.1. Limitations

A major limitation of our study is the fact that it is a retrospective, nonrandomized study with a relatively small number of patients, especially in the group treated by pacing. It should be emphasized that a highly selected population sent for treatment at the tertiary center was included, which could also affect some parameters. Moreover, selected inclusion criteria for the group treated with ASA may lead to the exclusion of complicated patients with potential suboptimal effect of ASA, who early underwent re-intervention, PM implantation or died. Also, followup, in particular its median, was nevertheless different between the groups.

## 5. Conclusion

The results of our study confirm that ASA is a safe and effective method of treatment of patients with obstructive HCM with sustained long-term effect. The long-term effect of DDD pacing on LVOTG was in our study more pronounced than the short term that was tested in the randomized clinical trials. So, the results of these studies cannot be probably regarded as determinative for the overall assessment of the effect of DDD pacing in the treatment of HCM. Cardiac pacing may be considered as a possible alternative to ASA or SME, especially in high-risk subjects and in patients with morphological substrate not suitable for ASA or SME. For definitive assessment of DDD, pacing position would be necessary to organize randomized trial with active treatment period exceeding 24 months or better prospective randomized trial focused on comparison of DDD pacing, ASA, or SME.

## Figures and Tables

**Table 1:** 

Parameter	ASA (*N* = 52)			DDD pacing (*N* = 24)			*P* value**
	Mean (SD)	Median (95% CI)	*P* value*	Mean (SD)	Median (95% CI)	*P* value*	
Age (years)	54.4 (13.69)	54.0 (50.0; 60.0)		50.0 (16.18)	49.0 (43.0; 58.0)		0.180
Follow up (months)	86.9 (23.14)	80.0 (74.0; 90.0)		101.2 (48.78)	109.5 (88.0; 132.0)		0.069
NYHA class							
Baseline	2.80 (0.478)	3.00 (3.0; 3.0)		2.65 (0.541)	3.00 (2.0; 3.0)		0.350
Final	1.66 (0.778)	1.25 (1.0; 2.0)		2.13 (0.576)	2.00 (2.0; 2.5)		0.005
Difference (final-baseline)	−1.13 (0.742)	−1.00 (−1.5; −1.0)	<0.001	−0.52 (0.561)	−0.50 (−1.0; 0.0)	<0.001	0.001
LVOTG (mmHg)							
Baseline	73.0 (37.82)	70.0 (54.0; 80.0)		81.7 (43.63)	78.5 (55.0; 100.0)		0.322
Final	23.6 (26.42)	10.0 (7.0; 25.0)		20.8 (21.19)	11.0 (6.0; 25.0)		0.960
Difference (final-baseline)	−49.4 (34.13)	−49.5 (−64.0; −34.0)	<0.001	−60.9 (47.51)	−53.5 (−74.0; −34.0)	<0.001	0.264
LVEF (%)							
Baseline	78.9 (8.50)	80.0 (78.0; 83.0)		70.1 (8.90)	70.0 (68.0; 75.0)		<0.001
Final	73.8 (9.94)	77.0 (70.0; 80.0)		63.0 (7.15)	65.0 (60.0; 70.0)		<0.001
Difference (final-baseline)	−5.1 (9.29)	−3.5 (−5.0; 0.0)	<0.001	−7.0 (8.93)	−6.5 (−10.0; 0.0)	0.001	0.312
LV Dd (mm)							
Baseline	41.8 (4.91)	42.0 (40.0; 44.0)		47.3 (5.66)	47.5 (44.0; 53.0)		<0.001
Final	47.0 (5.05)	48.0 (46.0; 49.0)		47.6 (5.49)	47.0 (44.0; 50.0)		0.874
Difference (final-baseline)	5.2 (5.91)	4.0 (2.0; 6.0)	<0.001	0.2 (5.57)	0.0 (−2.0; 5.0)	0.949	0.004
IVS (mm)							
Baseline	21.7 (3.96)	20.0 (20.0; 22.0)		23.4 (5.81)	22.0 (20.0; 26.0)		0.207
Final	14.2 (5.63)	13.0 (12.0; 15.0)		20.3 (7.21)	19.0 (17.0; 20.0)		<0.001
Difference (final-baseline)	−7.5 (6.19)	−7.8 (−9.5; −6.0)	0.001	−3.4 (4.01)	−3.0 (−5.0; −2.0)	0.001	<0.001

*P value: statistical significance of the parameter change in each group.

**P value: statistical significance of the difference in parameter change between both groups.
